# P35 Dermatomyositis with retinal involvement: a case series

**DOI:** 10.1093/rap/rkab068.034

**Published:** 2021-10-19

**Authors:** Wenona Barnieh, Jennifer Hannah, Aveen Connolly, Daniel Creamer, Patrick Gordon

**Affiliations:** 1 Department of Rheumatology, King’s College Hospital NHS Trust, London, United Kingdom; 2 Department of Dermatology, Guys and St Thomas’ NHS Trust, London, United Kingdom; 3 Department of Dermatology, King’s College Hospital NHS trust, London, United Kingdom

## Abstract

**Case report - Introduction:**

Dermatomyositis (DM) is a multisystem, autoimmune condition. It belongs to a spectrum of idiopathic inflammatory myopathies (IIMs) which can present with proximal muscle weakness or cutaneous lesions, but can also cause systemic disease including interstitial pneumonia, myocarditis, and inflammatory arthritis. Retinal involvement is not widely described. However, we present three cases with visual disturbance caused by retinal pathology – two of retinal vein occlusion, and one of retinal vasculitis. Two cases have anti-melanoma differentiation-associated gene 5 (anti-MDA-5), which may be a pathophysiological link to retinal microvascular damage.

**Case report - Case description:**

Patient 1:

A 43-year-old female with Anti-NXP-2 juvenile DM presented with bilateral visual loss. She was diagnosed with DM aged 6 when she presented with calcinosis, weakness, inflammatory arthritis and rash. Though disease was most active during adolescence, she has ongoing problematic calcinosis. ESR remains 26mm/hr. Fundoscopy showed scattered haemorrhages and macular oedema with no features of retinal vasculitis. Thrombophilia screens including antiphospholipid were negative. Bilateral branch retinal vein occlusions were treated with anti-VEGF injections. Post-treatment visual acuity is 6/6.

Patient 2:

A 46-year-old female with Anti-MDA-5 DM presented with monocular reduced visual acuity. She was diagnosed with a hemi-retinal vein occlusion. Thrombophilia screen showed she was antiphospholipid negative, but a heterozygote carrier of Factor V Leiden. She had moderately active disease at the time with evidence of organising pneumonia, and active Gottron’s papules. Prednisolone was increased, tacrolimus dosing was optimised and clopidogrel commenced. Later that year she was given rituximab for her organising pneumonia to which her skin showed good response. However, 8 months later she had a further central retinal vein occlusion with macular oedema treated with Anti-VEGF injections. Her subsequent visual acuity is 6/6.

Patient 3:

A 36-year-old male with Anti-MDA-5 DM with skin, joint and lung involvement was initially treated with IV cyclophosphamide. He then developed severe Raynaud’s, perniotic skin rash and vasculitic digital ulceration. He noted reduction in visual acuity. Fundoscopy revealed bilateral cotton wool spots and wide-field fundus fluorescein angiography showed microaneurysms. A diagnosis of a mild retinal vasculitis was made. He was admitted for epoprostenol, rituximab and 50mg prednisolone. Antiphopsholipid screen was negative. Vasculitic lesions on his fingers improved, and retinal abnormalities resolved. He has been on low-dose prednisolone without additional DMARDS for several years, with no recurrence of retinal disease in 10 years and skin has stabilised.

**Case report - Discussion:**

Retinal vein occlusion (RVO) is the second most common retinal vascular disease; it typically causes painless visual loss in older people above the age of 60. Obstruction of the retinal venous system is caused by thrombus, compression by an adjacent retinal artery or disease within the vein such as vasculitis. This leads to retinal ischaemia and elevated retinal vascular endothelial growth factor (VEGF) levels. VEGF increases the vascular permeability contributing to macular oedema. Vision loss occurs secondary to macular oedema or ischaemia. Treatment is with anti-VEGF injections and risk factor modification.

There is an association with thrombophillic disorders including antiphospholipid syndrome and hyperhomocysteinemia. Additional risk factors include increasing age, hypertension, hyperlipidaemia, glaucoma and diabetes. Cases of RVO have been described in dermatomyositis and other systemic autoimmune diseases. The exact mechanism is not fully understood but may be due to vasculopathy or microvascular damage.

Our cases were all under the age of 60 with no significant risk factors other than patient 2’s history of heterozygotic factor V Leiden, which only slightly increases the risk of blood clots. The simultaneously active inflammatory disease at the time and recurrent thrombosis whilst on clopidogrel adds suspicion of a vasculopathic component. All cases were anti-phospholipid negative.

Anti-MDA-5 disease is associated with complement-mediated microvascular damage leading to striking mucocutaneous manifestations. Lesional biopsies have demonstrated occlusive small and medium vessel vasculopathy with a type-1-interferon signature. Anti-NXP-2 has also been linked to GI-tract vasculitis and perforation in a JDM case series. It is plausible similar damage has occurred in the retinal vasculature of our cases. Active inflammation in the venules is classically seen in Behcet’s disease, but not typical of other connective tissue disease. Rather than a vasculitis, the two RVO cases may reflect a vasculopathy due to endothelial activation.

**Case report - Key learning points:**

The inflammatory process in DM can cause microangiopathic damage. This is evident in the skin lesions of MDA-5-positive disease, and is also postulated to be the pathophysiology behind complications such as rapidly progressive interstitial lung disease, which is most common for patients with anti-MDA-5 disease and carries a high mortality. A case series of GI perforation in NXP-2-positive JDM suggest that microangiopathy may be a feature of anti-NXP-2 disease also.

Retinal involvement in DM is rare, but there have been a few reports in the literature. Retinal vasculitis can also be caused by other systemic inflammatory diseases including Behcet’s, SLE, granulomatosus polyangiitis, sarcoidosis and multiple sclerosis. Differentials include ocular inflammatory disease (e.g., pars planitis), infectious disease (e.g., toxoplasmosis, cytomegalovirus, herpes simplex virus) or malignancy.

Retinopathy associated with DM usually completely resolves without complications but if left untreated, profound visual loss may result from macular haemorrhage or macular oedema leading to central scotomas. Potentially, a vasculopathic process with endothelial activation has propagated retinal vein occlusions in two of our cases. RVO can lead to permanent visual loss.

Early identification and treatment with anti-VEGF injections or immunosuppressants has prevented visual loss in our three patients. It remains unknown whether escalating immunosuppression in the two cases of RVO would reduce chances of recurrence. RVO is not in itself a rare disease, and it remains possible that simultaneous diagnoses with DM could be coincidental rather than pathologically directly linked, though the young age of all affected patients would be atypical. Traditional modifiable risk factors such as diabetes, hypertension and hyperlipidaemia should always be excluded and managed first.

It is important to be aware that patients with DM and other idiopathic inflammatory myopathies may be at increased risk of retinal complications and any new visual symptoms should be investigated appropriately.

